# Thymic adenocarcinoma accompanied by type A thymoma and pulmonary minimally invasive adenocarcinoma and harboring distinct gene alterations: A case report

**DOI:** 10.1097/MD.0000000000025254

**Published:** 2021-04-16

**Authors:** Yi-Wen Zheng, Lin-Lin Bai, Gui-Yang Jiang, Xu-Yong Lin, Yang Liu, Hong-Tao Xu

**Affiliations:** a Department of Pathology, the First Hospital and College of Basic Medical Sciences, China Medical University, Shenyang; b Department of Pathology, Shenyang 242 Hospital, Shenyang, China.

**Keywords:** *ARID1A*, *KMT2A*, mutation, thymic neoplasm, thymoma, TTF-1

## Abstract

**Rationale:**

Thymic adenocarcinoma is an extremely rare thymic carcinoma. The exact genetic alteration associated with thymic adenocarcinoma is unclear. Here, we report a case of thymic adenocarcinoma accompanied by type A thymoma and pulmonary minimally invasive adenocarcinoma (MIA).

**Patient concerns:**

A 53-year-old woman presented with multiple nodules in the mediastinum and lung. Thoracic computed tomography revealed nodules in the anterior superior mediastinum and anterior mediastinum near the right pericardium and ground-glass opacity (GGO) in the right superior lobe of the lung.

**Diagnosis:**

The tumor in the anterior superior mediastinum was diagnosed as primary thymic papillary adenocarcinoma. The tumor in the anterior mediastinum near the right pericardium was diagnosed as type A thymoma. The GGO of the right superior lobe of the lung was diagnosed as a MIA.

**Intervention:**

The patient underwent thoracoscopic mediastinal tumor resection and partial lobectomy in our hospital.

**Outcomes:**

The postoperative course was uneventful. The patient is alive and free of the disease for 22 months after diagnosis.

**Lessons:**

Thyroid transcription factor 1 (TTF-1) was positive in this case of thymic adenocarcinoma, which indicated that a thymic adenocarcinoma with TTF-1-positive may not necessarily be a metastasis of lung or thyroid adenocarcinoma. The positive staining of CD5 and CD117 can help us to confirm the thymic origin. Molecular genetic analysis indicated that these tumors harbored different mutations. The thymic adenocarcinoma and type A thymoma both had the mutation of *KMT2A*, but the mutation sites were different. *KMT2A* mutation may be a common genetic change in thymic tumorigenesis. The genetic alterations disclosed in this study will help expand the understanding of thymic tumors.

## 1 Introduction

Thymic adenocarcinoma is an extremely rare thymic carcinoma. It accounts for only 0.48% (29/6097) of thymic epithelial neoplasms.^[1]^ According to the WHO classification of tumors of thymus, the thymic adenocarcinoma was classified as papillary adenocarcinoma, thymic carcinoma with adenoid cystic carcinoma-like features, mucinous adenocarcinoma, and adenocarcinoma, not otherwise specified.^[2,3]^ The mean age of patients was in their 50 s. The male-to-female ratio was about 2:1.^[2]^ The etiology is unclear due to thymic adenocarcinoma is very rare. Thymic papillary adenocarcinoma was considered associated with type A or AB thymomas.^[4,5]^ Mucinous adenocarcinoma may arise in multilocular thymic cysts.^[3]^ But, the exact genetic alteration associated with thymic adenocarcinoma is unclear. Here, we report a case of thymic adenocarcinoma accompanied by type A thymoma and pulmonary minimally invasive adenocarcinoma (MIA), and analyzed the genetic alterations of them using next-generation sequencing (NGS).

## 2 Case presentation

### 2.1 Ethic approval

This study was approved by the institutional review board of China Medical University for human studies. The ethical board approval number is LS[2018]016. A written informed consent was obtained from the patient for the publication of this case report and accompanying images.

### 2.2 Clinical history

**Figure 1:**
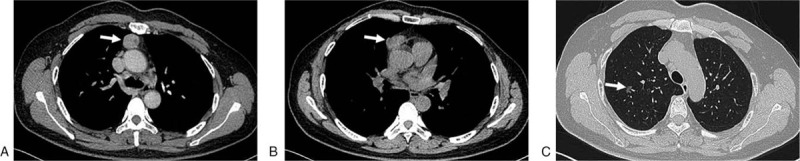
Thoracic computed tomography examination. (A) A mass in the anterior superior mediastinum (arrow). (B) A mass in the anterior mediastinum near the right pericardium (arrow). (C) A ground-glass opacity, about 1.0 cm in diameter, in the right superior lobe of the lung (arrow).

A 53-year-old woman presented in December 2018 with multiple nodules in the mediastinum and lung, with occasional right-chest discomfort. The patient had no cough, expectoration, and fever. Thoracic computed tomography revealed nodules in the anterior superior mediastinum (3.0 × 2.0 cm) and anterior mediastinum near the right pericardium (4.0 × 3.0 cm) and ground-glass opacity (GGO, 1.0 cm) in the right superior lobe of the lung (Fig. 1). Her laboratory examination showed no abnormalities. Thoracoscopic mediastinal tumor resection and partial lobectomy were performed.

### 2.3 Morphological findings

Macroscopically, the tumor of anterior superior mediastinum had a relatively clear border. The cut surface of the tumor was cystic and solid, containing a large amount of fine gray-white papillary substance. The tumor in the anterior mediastinum near the right pericardium was solid and gray-white, with a partial capsule. The GGO of the right superior lobe of the lung was dark gray and its boundary was not clear with the surrounding lung tissue.

**Figure 2:**
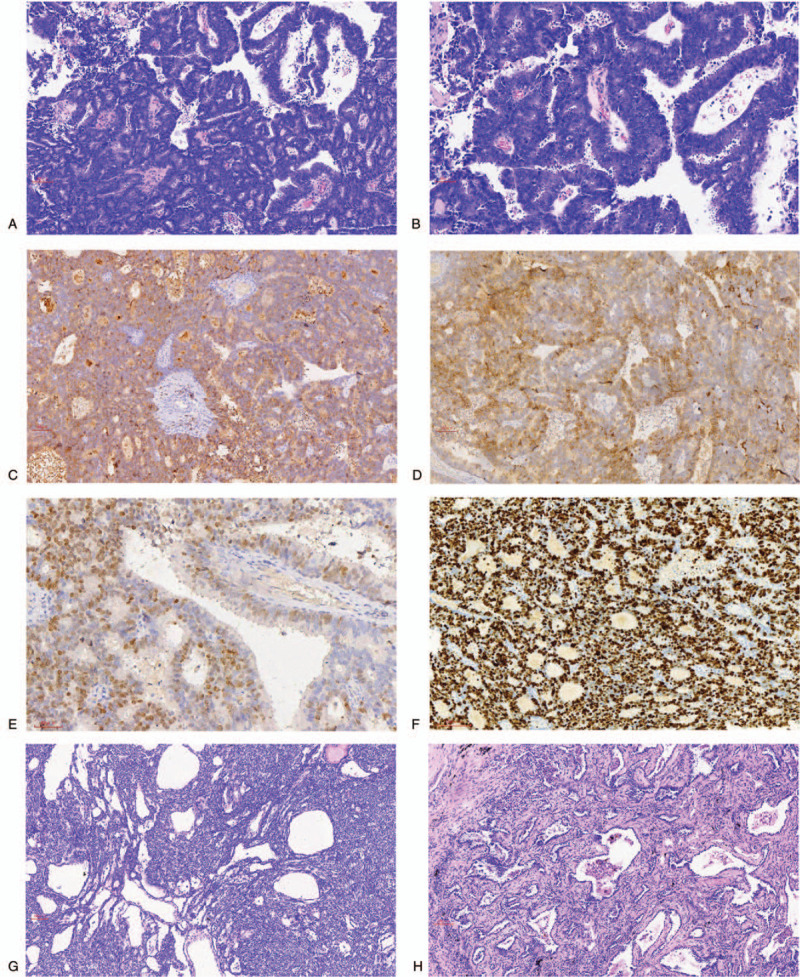
Pathologic findings. (A) In the tumor in the anterior superior mediastinum, the papillary structures were covered by columnar tumor cells, which formed a complex glandular structure or cribriform (hematoxylin and eosin [HE]; original magnification, ×100). (B) A magnified version of part of figure **A** (middle-upper). The nuclei of tumor cells were enlarged and hyperchromatic and had many mitotic figures (HE; original magnification, ×200). (C and D**)** The tumor cells were positive for CD117 (C) and CD5 (D) staining (immunohistochemistry; original magnification, × 100). (E) Some tumor cells were positive for TTF-1 staining (immunohistochemistry; original magnification, ×200). (F) The Ki-67 index was >80% (immunohistochemistry; original magnification, ×100). (G) The tumor in the anterior mediastinum near the right pericardium was a type A thymoma (HE; original magnification, ×100). (H) The ground-glass opacity in the right superior lobe of the lung was found to be a minimally invasive adenocarcinoma (HE; original magnification, ×100).

The resected tumor tissue was fixed with 10% neutral-buffered formalin, embedded into paraffin blocks, and cut into 4-μm sections. The tissue sections were stained with hematoxylin and eosin for histological evaluation. Microscopically, the tumor of anterior superior mediastinum was cystic and solid with complex papillary structures. The fibrovascular axis was covered by columnar tumor cells, forming a complex glandular structure or cribriform. The tumor cell nuclei were enlarged and hyperchromatic, with many mitotic figures. Necrosis was observed (Fig. 2A and B).

### 2.4 Immunohistochemical findings

Immunohistochemical staining was performed with ready-to-use primary antibodies, which were purchased from Maixin, Fuzhou, China. After incubation with the primary antibody, the presence of antibodies was assessed using the streptavidin–peroxidase method. Positive and negative controls were used accordingly to prevent false positivity and negativity. The tumor tissue of anterior superior mediastinum was strongly positive for CD5 and CD117 (Fig. 2C and D), which indicated a thymic epithelial origin. Furthermore, the tumor cells were positive for cytokeratin (CK) 19; partially positive for thyroid transcription factor-1 (TTF-1) (Fig. 2E); and negative for Pax-8, Napsin A, CDX-2, SATB2, and terminal deoxynucleotidyl transferase (TdT). The Ki-67 index was >80% (Fig. 2F). Therefore, this tumor was diagnosed as primary thymic papillary adenocarcinoma.

The tumor in the anterior mediastinum near the right pericardium was pathologically diagnosed as type A thymoma (Fig. 2G). The GGO of the right superior lobe of the lung was diagnosed as a MIA (Fig. 2H). The patient did not receive any postoperative adjuvant therapies and is alive and free of the disease for 22 months after diagnosis.

### 2.5 Molecular genetic findings

**Figure 3:**
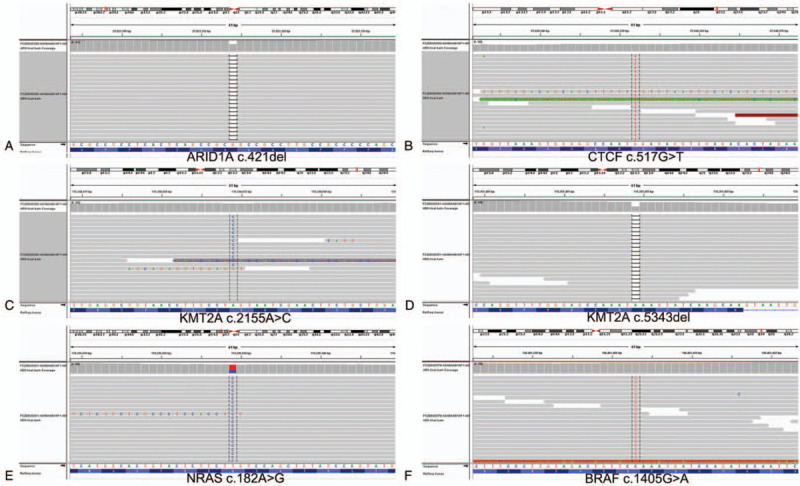
Molecular genetic studies using next-generation sequencing. (A–C**)***ARID1A* c.421del (p.A141Pfs∗91) (A), *CTCF* c.517G>T(p.G173∗) (B), and *KMT2A* c.2155A>C (p.S719R) (C) mutations were detected in the thymic adenocarcinoma. (D and E) The type A thymoma showed *KMT2A* c.5343del(p.V1782Yfs∗38) (D) and *NRAS* c.182A>G (p.Q61R) (E) mutations. (F) The *BRAF* c.1405G>A (p.G469R) mutation was detected in the minimally invasive adenocarcinoma of the lung.

To further investigate whether these tumors had similar genetic alterations, NGS analysis was performed. It revealed that there were distinct genetic mutations. The thymic adenocarcinoma harbored many mutations, including *ARID1A* c.421del(p.A141Pfs∗91) (Fig. 3A), *CTCF* c.517G>T(p.G173∗) (Fig. 3B), *KMT2A* c.2155A>C(p.S719R) (Fig. 3C), *BAP1* c.673G>A(p.D225N), *ERBIN* c.2900C>G(p.S967C), *LZTR1* c.2216C>T(p.S739L), *POLE* c.52G>C(p.E18Q), and *SRC* c.1334A>C(p.K445T) (Supplemental Figure 1). The type A thymoma showed *KMT2A* c.5343del(p.V1782Yfs∗38) and *NRAS* c.182A>G(p.Q61R) mutations (Fig. 3D and E). The MIA revealed a *BRAF* c.1405G>A(p.G469R) mutation (Fig. 3F).

## 3 Discussion

Thymic adenocarcinoma is extremely rare. There are only a few sporadic reports.^[4–12]^ Till now, only about 60 cases of primary thymic adenocarcinoma have been reported.^[5,8,11]^ Because of its low incidence, it is necessary to differentiate it from other metastatic adenocarcinoma, such as lung adenocarcinoma, colorectal cancer, thyroid cancer, and so on. Immunohistochemical staining is very helpful in differential diagnosis. Thymic adenocarcinoma often positive for CD5 and/or CD117, which are negative in adenocarcinomas of other sites. In addition, some tissue specific markers, such as TTF-1 and Napsin A for lung adenocarcinoma, CDX-2 and SATB2 for colorectal cancer, PAX-8 and TTF-1 for thyroid cancer, are often negative in thymic adenocarcinoma.^[8]^ But, in this case, we for the second time reported that TTF-1 was positive in some tumor cells, which confirmed previous report in primary thymic papillary adenocarcinoma.^[5]^ Therefore, a thymic adenocarcinoma with TTF-1 positive may not necessarily be a metastasis of lung or thyroid adenocarcinoma. Primary thymic adenocarcinoma cannot be excluded only by TTF-1-positive alone. The positive staining of CD5 and CD117 can help us to confirm the thymic origin.

Thymic papillary adenocarcinoma is often accompanied by type A or AB thymoma or multilocular thymoma cysts with transition zone.^[4,5,13–16]^ Therefore, thymic papillary adenocarcinoma was considered arise in type A or AB thymomas. Here, the thymic adenocarcinoma and type A thymoma were located in different regions, without a visible connection. NGS analysis revealed distinct genetic alterations among these tumors. This was for the first time examined and compared the genetic alterations of thymic tumors and MIA in a same patient. Although the thymic adenocarcinoma and type A thymoma both had the mutation of *KMT2A*, the mutation sites were different. So, these tumors were independent primary tumors. Previous studies identified many genetic aberrations in thymic adenocarcinoma, including a homozygous deletion at the *HLA* locus and *KRAS* mutation.^[6,7,17]^ But due to the small number of cases, no genetic changes with high incidence were found in thymic adenocarcinomas. *KMT2A-MAML2* gene fusion was reported in 4% thymomas (10 of 242), and was considered specific to type B2 and B3 thymomas.^[18]^ Here, we showed the mutation of *KMT2A* can occur in thymic adenocarcinoma and type A thymoma, suggesting that *KMT2A* mutation may not specific to type B2 and B3 thymomas, but a common genetic change in thymic tumorigenesis. *ARID1A* mutation was found in a B2 thymoma previously.^[18]^ Here, we indicated it also can occur in thymic adenocarcinoma. Many other mutations were first detected in this case. Their function and significance need further clarification.

## Acknowledgments

The authors thank the technical assistance in genetic analysis from Dr. Dadong Ding and Dr. Peng Yang from Nanjing Geneseeq Technology Inc.

## Author contributions

**Conceptualization:** Hong-Tao Xu.

**Funding acquisition:** Hong-Tao Xu.

**Methodology:** Yi-Wen Zheng, Lin-Lin Bai, Gui-Yang Jiang, Xu-Yong Lin, Yang Liu.

**Writing – original draft:** Yi-Wen Zheng, Hong-Tao Xu.

**Writing – review & editing:** Gui-Yang Jiang, Xu-Yong Lin, Hong-Tao Xu.

## Supplementary Material

Supplemental Digital Content
