# Supplementary material for: Thymic adenocarcinoma accompanied by type A thymoma and pulmonary minimally invasive adenocarcinoma and harboring distinct gene alterations: A case report
Source: Medicine (Baltimore). 2021 Apr 16;100(15):e25254. doi: 10.1097/MD.0000000000025254 (PMC8052068; doi:10.1097/MD.0000000000025254)

**Supplemental Figure 1 Other genetic mutations in this case of thymic adenocarcinoma.**

**A–E.** *BAP1* c.673G>A(p.D225N) (A), *ERBIN* c.2900C>G(p.S967C) (B), *LZTR1* c.2216C>T(p.S739L) (C), *POLE* c.52G>C(p.E18Q) (D), and *SRC* c.1334A>C(p.K445T) (E) mutations were also detected in this case of thymic adenocarcinoma.


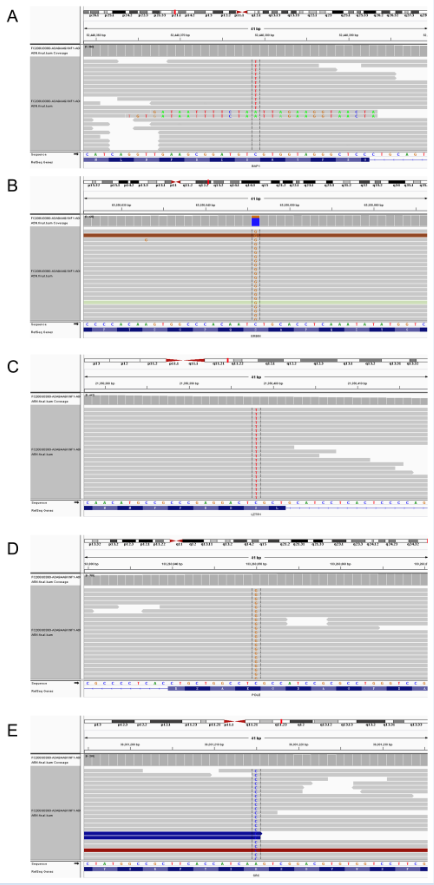

Supplement: Supplemental Digital Content [file medi-100-e25254-s001.doc]
